# Detection of Prions in Brain Homogenates and CSF Samples Using a Second-Generation RT-QuIC Assay: A Useful Tool for Retrospective Analysis of Archived Samples

**DOI:** 10.3390/pathogens10060750

**Published:** 2021-06-13

**Authors:** Tibor Moško, Soňa Galušková, Radoslav Matěj, Magdalena Brůžová, Karel Holada

**Affiliations:** 1 Institute of Immunology and Microbiology, First Faculty of Medicine, Charles University, 12800 Prague, Czech Republic; Sona.Galuskova@lf1.cuni.cz (S.G.); khola@lf1.cuni.cz (K.H.); 2 Department of Pathology and Molecular Medicine, Third Faculty of Medicine, Charles University and Thomayer University Hospital, 14059 Prague, Czech Republic; radoslav.matej@ftn.cz (R.M.); magdalena.smetakova@ftn.cz (M.B.); 3 Department of Pathology, Third Faculty of Medicine, Charles University and University Hospital Kralovske Vinohrady, 10034 Prague, Czech Republic

**Keywords:** RT-QuIC assay, prion diseases, CJD, Creutzfeldt-Jakob disease, archived sample

## Abstract

The possibilities for diagnosing prion diseases have shifted significantly over the last 10 years. The RT-QuIC assay option has been added for neuropsychiatric symptoms, supporting biomarkers and final *post-mortem* confirmation. Samples of brain homogenates used for final diagnosis, archived for many years, provide the possibility for retrospective studies. We used a second-generation RT-QuIC assay to detect seeding activity in different types of sporadic and genetic prion diseases in archival brain homogenates and *post-mortem* CSF samples that were 2 to 15 years old. Together, we tested 92 archival brain homogenates: 39 with definite prion disease, 28 with definite other neurological disease, and 25 with no signs of neurological disorders. The sensitivity and specificity of the assay were 97.4% and 100%, respectively. Differences were observed in gCJD E200K, compared to the sporadic CJD group. In 52 *post-mortem* CSF samples—24 with definite prion disease and 28 controls—we detected the inhibition of seeding reaction due to high protein content. Diluting the samples eliminated such inhibition and led to 95.8% sensitivity and 100% specificity of the assay. In conclusion, we proved the reliability of archived brain homogenates and *post-mortem* CSF samples for retrospective analysis by RT-QuIC after long-term storage, without changed reactivity.

## 1. Introduction

Prion diseases, despite being rare, are invariably fatal. Sporadic Creutzfeldt-Jakob disease (sCJD), the most common human prion disease, belongs to the group of transmissible spongiform encephalopathies (TSEs) [1]. Aside from sporadic etiology, TSEs may have a genetic predisposition background, with mutations in the prion protein gene making the protein prone to misfolding and aggregation. Other genetic prion diseases include genetic CJD (gCJD), Gerstmann-Sträussler-Scheinker disease (GSS), and Fatal familial insomnia (FFI) [2]. Misfolding of the prion protein occurs at the beginning of the disease, regardless of the origin. This event is common to all prion diseases, and does not play a role if it arises stochastically (sporadic form), from a genetic predisposition, or enters the body from the outside (transmissible/iatrogenic form). Misfolded prion protein (PrP^Sc^) represents the only specific TSE marker, and its detection is used for final confirmation of the disease by immunohistochemistry and western blot in *post-mortem* brain samples. Clinical diagnosis of TSEs is based mainly on characteristic neuropsychiatric symptoms, supported by results of EEG tests, MRI scans, and diagnostic biomarkers, such as the level of 14-3-3 and total Tau protein in CSF [3]. The *ante-mortem* options for direct detection of prions are limited, due to low levels of PrP^Sc^ in peripheral tissues. However, the implementation of prion amplification technology in the diagnostics of TSEs, with which the misfolded protein conformation can be detected with very high sensitivity, has changed the situation fundamentally. Prions can be amplified exponentially in vitro by these methods. During the reaction, a conformational change of the soluble PrP occurs, leading to polymerization and the subsequent formation of aggregates. This mechanism is used in methods such as PMCA (protein misfolding cyclic amplification) or RT-QuIC assay [4,5,6,7]. An improved version of the RT-QuIC assay, represented as the second-generation RT-QuIC assay, has recently been described, and is characterized by increased speed and high sensitivity [8,9,10,11]. The RT-QuIC method is able to detect the presence of a femtogram of PrP^Sc^ in a CSF or nasal brushing sample, allowing for diagnosis of the disease at its beginning [12,13]. This opens the possibility of early administration of treatment, which may be ineffective when administrated later (i.e., when severe neurological symptoms have already appeared). The sensitivity and specificity of the method are respectable. According to various studies, they range from 73% to 97% and from 99% to 100%, respectively [12]; however, the sensitivity is not 100% and there have been several cases of CJD or genetic forms of prion diseases that were not detected as positive in various studies [13,14]. The reason why these cases were not reliably detected is not completely understood.

In our laboratory, we introduced the second-generation RT-QuIC assay, in order to improve the diagnostics of prion diseases at the National Reference Laboratory for Diagnostics of Human TSE/CJD. For internal validation of the method, we utilized archived samples to verify that RT-QuIC fulfills the requirements of the diagnostic test in our laboratory conditions. Together, we analyzed 144 brain homogenate (BH) or CSF samples, of which 39 BH and 24 CSF samples were from TSE patients, 28 BH and 28 CSF samples were controls with other neurodegenerative diseases (OND), and 25 BH samples were from corneal donor controls without any neurodegenerative disorder. We have focused on archived BH and CSF samples, due to their possible utilization in future retrospective studies. The selection of the samples corresponded to all sporadic forms of CJD, and to the representation of genetic prion diseases appearing in the Czech Republic.

## 2. Results

### 2.1. Establishment of the Second-Generation RT-QuIC Assay

**Figure 1:**
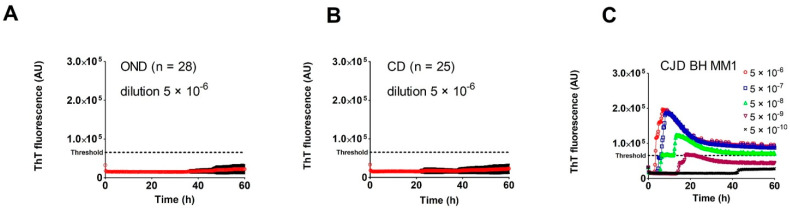
Establishment of the RT-QuIC assay threshold for *post-mortem* brain homogenates: (**A**) The results of the analysis of the control brain homogenate samples (dilution 5 × 10^−6^) of patients with other neurological diseases (OND, n = 28). The red line represents the arithmetic mean of ThT fluorescence (expressed as arbitrary units AU) at each time point, along with the standard deviation (black bars). The threshold was calculated as the arithmetic mean of maximal ThT fluorescence + 5 SD; (**B**) Analysis of control brain homogenate samples (dilution 5 × 10^−6^) of corneal donors (CD, n = 25); (**C**) Typical results of the second-generation RT-QuIC assay with serial dilutions of control sCJD brain homogenate (sCJD MM1). The assay gave a positive result for the brain homogenate diluted by 5 × 10^−9^.

We purified and refolded the rSHa PrP(90–231) substrate at the quality and quantity required for the second-generation RT-QuIC assay. The yield of the recombinant protein was 45–60 mg per purification. SDS-PAGE with Coomassie staining and immunoblotting confirmed the correct size of rSHa PrP (90–231): ~17 kDa (not shown). The quality control assay showed a robust positive RT-QuIC response for 5 × 10^−6^–5 × 10^−9^ dilutions of control sCJD MM1 BH and lack of spontaneous aggregation. To compare buffers for BHs, we prepared BH samples in buffer used for RT-QuIC (100 mM Tris-Cl, pH 8.0; 150 mM NaCl; 13 mM EDTA; 12 mM sodium deoxicolate; 0.5% IGEPAL CA-630) and in buffer used for western blot in our laboratory (see methods). The RT-QuIC assay results showed that the buffer used for western blot can be used for RT-QuIC, and the type of detergent and the absence of EDTA did not affect the assay. We tested BH dilutions from 5 × 10^−5^ to 5 × 10^−12^, which have been used in RT-QuIC studies with similar results (data not shown). The calculated thresholds for BH and CSF samples estimated using OND controls were 65,413 AU and 33,166 AU, respectively. The BH threshold calculated using corneal donors was 67,672 AU, almost identical to the OND BH threshold (Figure 1A,B). The calculated threshold for CSF diluted OND samples (76,436 AU) was more than twice higher than for undiluted CSF (not shown). As a standard positive control, we used freshly prepared sCJD MM1 BH in dilutions of 5 × 10^−6^ up to 5 × 10^−10^. The seeding activity in the positive control was regularly detected in a 5 × 10^−9^ dilution (Figure 1C), corresponding to a final dilution of BH in the reaction mix of 10^−11^.

### 2.2. Analysis of Archived Brain Homogenates Using RT-QuIC

**Figure 2:**
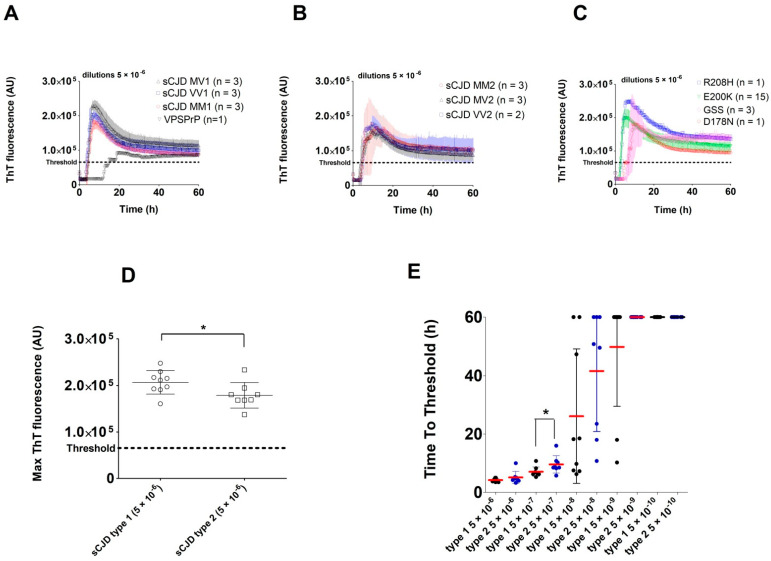
RT-QuIC analysis of archived brain homogenates of patients with sporadic and genetic prion diseases: (**A**) Kinetics of RT-QuIC reaction comparing samples of sCJD type 1 group (MM, VV and MV) and Variably Protease Sensitive Prionopathy case (VPSPrP) BHs (dilution 5 × 10^−6^). The VPSPrP sample had notably lower max ThT fluorescence and longer time to threshold; (**B**) Kinetics of RT-QuIC reaction comparing samples of sCJD type 2. group (MM, VV, and MV) BHs (dilution 5 × 10^−6^). One case of sCJD VV2 gave a negative result and was omitted from the mean calculation; (**C**) Kinetics of RT-QuIC reaction in the group of genetic prion diseases (gCJD E200K cases, R208H, D178N) and results of GSS BH samples. Kinetics of GSS BHs showed increased variability of max ThT fluorescence and time to threshold; (**D**) Comparison of max ThT fluorescence values of sCJD type 1 and 2 BHs diluted 5 × 10^−6^ (* *p* < 0.05); (**E**) Comparison of time to threshold values between groups of sCJD type 1 and 2 BHs (* *p* < 0.05). Arithmetic mean values with standard deviation are shown. A time of 60 h was assigned when the threshold was not reached.

Seeding activity was detected in all archived BH samples with confirmed TSE, except for one case of sCJD VV2. No control OND or corneal donors BH gave positive results (Figure 1A,B). The mean maximal ThT fluorescence of glycotype 1 sCJD BHs was significantly higher (207063 AU vs. 179126 AU, *p* < 0.05; Figure 2D), and their time to threshold value at a dilution of 5 × 10^−7^ was shorter (*p* < 0.05; Figure 2E) than the respective values of glycotype 2 sCJD BHs. Differences within the 129 polymorphism group in maximal ThT fluorescence and time to threshold values were not evaluated, due to the small number of analyzed samples in the individual groups. The samples from the sCJD group were clearly identifiable as positive in all four replicates at 5 × 10^−6^ sample dilution, and were positive at a dilution of BH up to 5 × 10^−8^ or 5 × 10^−9^ (Figure 2E).

**Figure 3:**
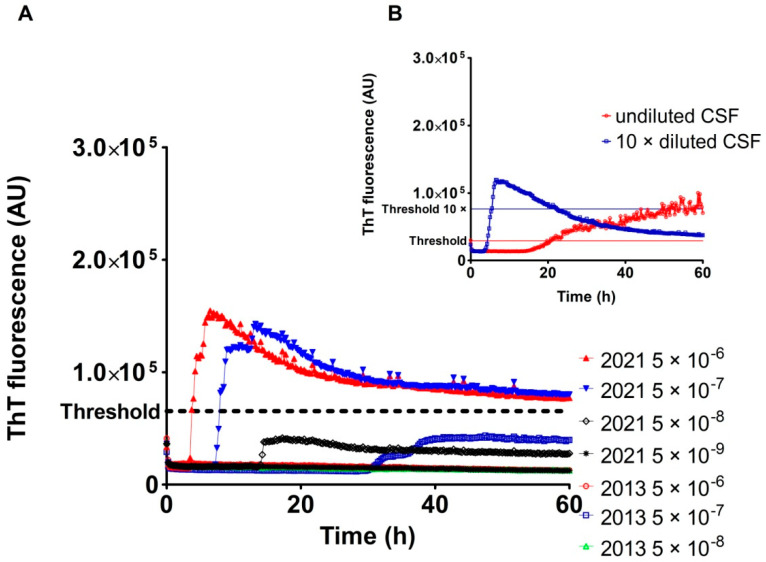
Analysis of the sCJD VV2 case with RT-QuIC negative archived brain homogenate: (**A**) Kinetics and serial dilutions of BH (sCJD VV type 2). The RT-QuIC assay, using archived BH (2013), provided negative results. Freshly prepared BH (2021) showed positive results in dilutions of 5 × 10^−6^ and 5 × 10^−7^; (**B**) Analysis of the corresponding archived CSF, confirming the presence of prion seeding activity.

The one sCJD VV2 case gave negative results in three independent RT-QuIC assays utilizing two different archived BH aliquots. The negative results of the RT-QuIC assay of the case were unequivocal with characteristic spongiform changes and presence of proteinase K-resistant prion protein, visualized both by immunohistochemistry and western blot at the time of sampling (not shown). RT-QuIC analysis of the corresponding archived CSF samples confirmed the presence of prions (Figure 3B); however, the shape of the curve representing the ThT fluorescence signal was unusual, suggesting either the potential presence of inhibitors or spontaneous aggregation. The 10× dilution of the CSF sample led to clear positivity with a typical curve shape (Figure 3B). To demonstrate that the negative RT-QuIC results were likely caused by BH degradation during long-term storage, we prepared a fresh BH from frozen brain tissue. Fresh BH showed clear RT-QuIC positivity in the dilutions of 5 × 10^−6^ and 5 × 10^−7^ (Figure 3A). The max ThT fluorescence was lower than in other tested sCJD BHs, but clearly above the threshold.

**Figure 4:**
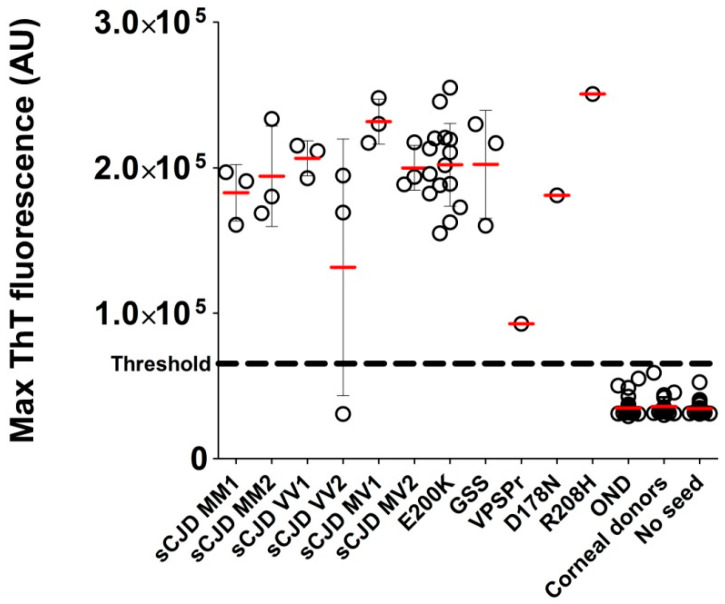
Average of the max ThT fluorescence of the archived brain homogenates in all patient groups. Average fluorescence maxima from quadruplicates of all RT-QuIC-tested BHs diluted by 10^−6^. Bars represent the arithmetic mean of the group and error bars represent the standard deviation. The threshold is indicated by a dashed line. All control samples are below the threshold of the assay. One case of sCJD VV2 was diagnosed as negative by RT-QuIC. The VPSPr case had lower max ThT fluorescence; however, it was clearly above the threshold.

The VPSPr BH analyzed by RT-QuIC revealed clear positivity in all four wells, but the max ThT fluorescence at a dilution of 5 × 10^−6^ (92753 AU) was notably lower than in the other TSE cases (Figure 4) and the time to threshold was markedly longer (Figure 2A).

**Figure 5:**
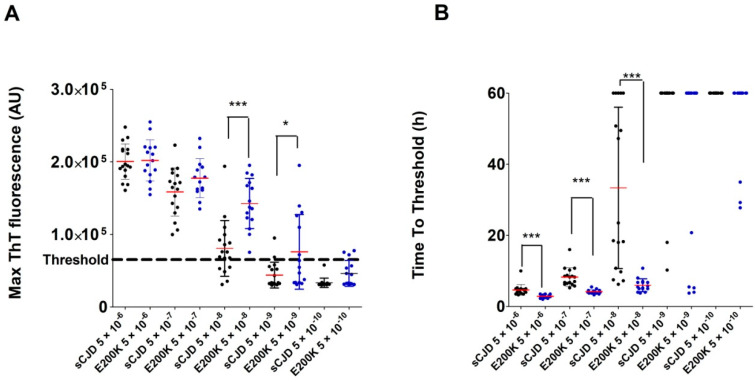
Comparison of the RT-QuIC analysis of sCJD- and gCJD E200K-archived brain homogenates: (**A**) Max ThT fluorescence of sCJD (n = 17) and gCJD E200K (n = 15) BHs in 10^−6^–10^−10^ dilutions (* *p* < 0.05, *** *p* < 0.0001); (**B**) Time to threshold of sCJD (n = 17) and gCJD E200K (n = 15) BHs in 10^−6^–10^−10^ dilutions (*** *p* < 0.0001). Red bars represent the arithmetic mean of the group and error bars represent the standard deviation. The threshold is indicated by a dashed line. A time of 60 h was assigned when the threshold was not reached.

The RT-QuIC analysis of all gCJD E200K-archived BHs (n = 15) led to well-shaped curves and short time to threshold values (Figure 2C). The E200K BHs produced significantly higher max ThT fluorescence than sCJD BHs at dilutions 5 × 10^−8^ and 5 × 10^−9^ (Figure 5A). The mean time to threshold was also significantly shorter for E200K BHs than for sCJD BHs (*p* < 0.0001, Figure 5B). The codon 129 polymorphism (MM, MV, VV) in the E200K group did not have any significant effect on max ThT fluorescence or time to threshold values (not shown). Archived BH samples of GSS P102L patients (n = 3) were all positive by RT-QuIC, with max ThT fluorescence values similar to those of the sCJD or E200K groups (Figure 4). Two additional BHs of gCJD cases with mutations D178N (MV2) and R208H (VV2) were also clearly positive (Figure 2C and Figure 4).

Taken together, the analysis of the archived *post-mortem* BH samples of patients with a definite diagnosis of TSE (n = 39), OND patients (n = 28), and normal corneal donors (n = 25) led to 100% specificity and 97.4% sensitivity of the second-generation RT-QuIC assay.

### 2.3. Analysis of Archived CSF Samples with the RT-QuIC Assay

**Figure 6:**
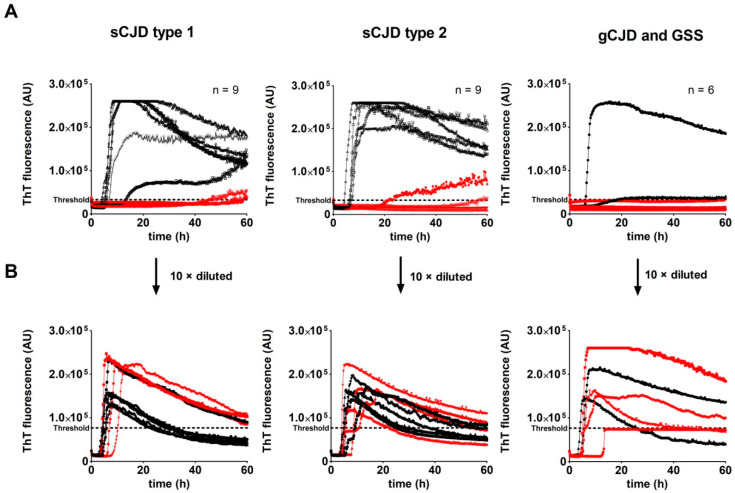
Comparison of the RT-QuIC assay kinetics utilizing undiluted and diluted archived *post-mortem* CSF samples of sCJD and gCJD patients. Three samples of each sCJD type (MM1, MV1, VV1, MM2, MV2, VV2) and of gCJD E200K and GSS P102L were analyzed (n = 24): (**A**) Kinetics of undiluted CSF samples. Red curves represent samples with an unusual curve shape or that do not cross the threshold; (**B**) Kinetics of CSF samples diluted 10 times. Red and black curves represent the same samples as in A. After CSF dilution, the kinetics of RT-QuIC reactions had a typical shape and all samples crossed the threshold, except for one GSS sample. The threshold at A and B was calculated using values of undiluted and diluted CSF samples of the OND controls (n = 28), respectively.

We analyzed three archived CSF samples of each sCJD type (MM1, MV1, VV1, MM2, MV2, and VV2) and three of gCJD E200K and GSS P102L (n = 24). Control CSF samples of OND patients (n = 28) were used for determination of the threshold (33,166 AU). The RT-QuIC assay with undiluted CSF samples of TSE patients provided unconvincing results. Of the 24 samples, only 13 samples gave a typical response curve (Figure 6A).

**Figure 7:**
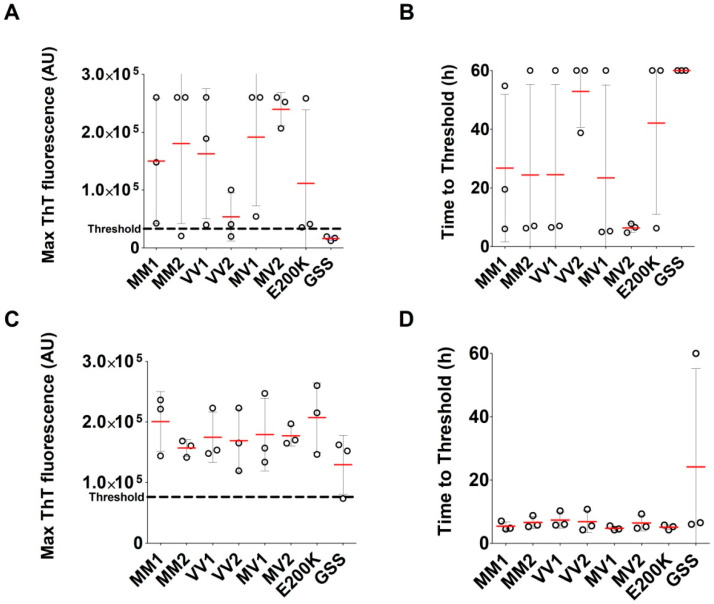
Comparison of the RT-QuIC analysis of archived *post-mortem* CSF samples of patients with sporadic and genetic TSEs. Three samples of each sCJD type (MM1, MV1, VV1, MM2, MV2, VV2) and of gCJD E200K and GSS P102L were analyzed (n = 24): (**A**) Max ThT fluorescence of undiluted CSF samples in different types of TSEs; (**B**) Corresponding time to threshold of undiluted CSF samples; (**C**) Max ThT fluorescence of diluted CSF samples in different types of TSEs. Only one GSS sample did not reach the threshold; (**D**) Corresponding time to threshold of diluted CSF samples. The differences in measured values among the groups were not statistically significant. Red bars represent the arithmetic mean of the group (n = 3) and error bars represent standard deviation. The threshold is indicated by a dashed line. A time of 60 h was assigned when the threshold was not reached.

**Figure 8:**
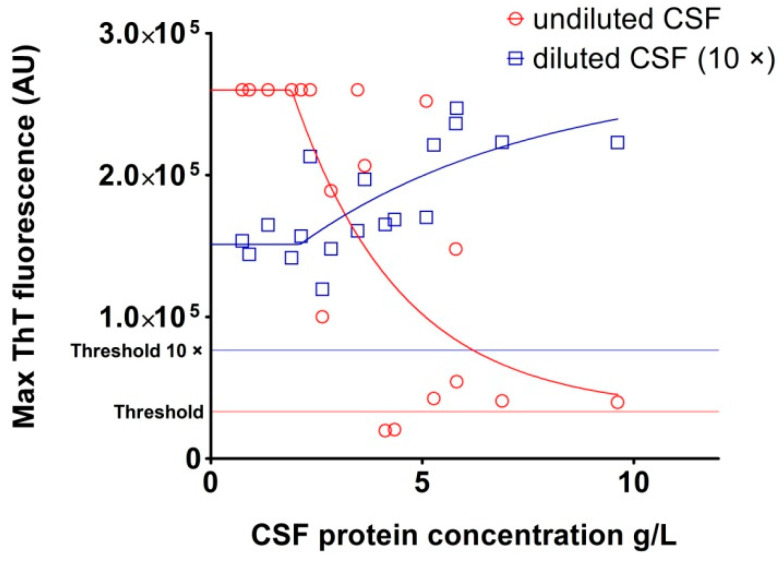
Relationship between maximal ThT fluorescence and protein concentration of *post-mortem* sCJD CSF samples. The set of *post-mortem* sCJD samples (n = 18) was analyzed by second-generation RT-QuIC undiluted (red circles) and 10× diluted (blue squares). The protein concentrations of the samples were determined by the BCA method. The CSF samples with a protein concentration above 2.5 g/L inhibited the RT-QuIC reaction. Dilution of the samples eliminated the inhibiting effect on the assay.

The rest of the samples had a low max ThT fluorescence value with an unusually long time to threshold, and five samples were negative (Figure 7A,B). The sensitivity of the assay with undiluted archived CSF samples was 79.2%. The protein content of archived *post-mortem* CSF samples was heterogeneous and notably higher than the physiological CSF protein concentration (3.8 ± 2.3 g/L vs. 0.2–0.4 g/L), suggesting the possible presence of RT-QuIC inhibitors. Repetition of the analysis with 10 times diluted CSF samples provided much more convincing results. The threshold, calculated using diluted OND CSF samples, increased to 76,436 AU. Of the 24 TSE CSF samples, 23 produced typical positive curves (Figure 6B). The max ThT fluorescence of the samples with previous poor response substantially increased and the time to threshold dramatically shortened (Figure 7C,D). Only one GSS sample was assessed as negative, with response just below the threshold. The sensitivity of the assay with diluted archived CSF samples increased to 95.8%. The dependence of RT-QuIC results on CSF protein concentration is shown in Figure 8. The samples with a protein content above 2.5 g/L exhibited inhibitory effects in the assay. The dilution of the CSF samples prevented RT-QuIC inhibition without compromising the assay sensitivity.

## 3. Discussion

The second-generation RT-QuIC, also known as the IQ assay (Improved QuIC), is a version of the assay with increased sensitivity and significantly shorter time of detection [10]. Almost all human studies utilizing second-generation RT-QuIC have used CSF as the analyte [8,9,10,11,15,16,17,18,19,20,21]. In our retrospective study, we evaluated the second-generation RT-QuIC assay for the analysis of archival *post-mortem* BH and CSF samples within a TSE patient cohort typical of the Czech Republic [22,23]. In addition to samples of different types of sCJD, we included a large group of the most common gCJD in the Czech Republic, E200K. Other studies including gCJD with mutations D178N or R208H and GSS with P102L mutation are comparatively rare [24]. The archived BH samples were originally prepared for confirmation of the presence of PK-resistant PrP^Sc^ by western blot. The samples were stored as 10% BH in lysis buffer containing detergents at −80 °C for 2 to 15 years. To estimate the diagnostic accuracy of the assay, we selected two different control groups, in order to prevent misinterpretation of the results. A group of patients with other neurodegenerative disorders was used as the most relevant differential diagnostic group and healthy corneal donors served as a tool for the identification of potential OND false positivity; however, both control groups provided similar data, with nearly identical calculated threshold values and without false positive results. This is an important observation, suggesting that, in the case of archival BH samples, negative specimens have the same background whether they come from healthy controls or neuropathologically proven neurodegeneration. All tested BH samples with definite diagnosis of TSE, except for one sCJD VV2 case, were RT-QuIC positive. Two different aliquots of the sCJD VV2 archived BH were repeatedly RT-QuIC negative, but the archived CSF gave a positive result. The false-negative BH was stored for 7 years; however, other much older samples tested positive, with no apparent effect of storage time on the test results. The archival BH was positive for the presence of PK-resistant PrP^Sc^ by western blot in 2013, but not in 2021, suggesting that the negative RT-QuIC result was, indeed, caused by sample degradation. Freshly prepared sCJD VV2 BH showed positivity both in RT-QuIC and western blot. The RT-QuIC assay of 92 *post-mortem* archival BH samples showed 97.4% sensitivity and 100% specificity, comparable to previously published results [12]. Our data suggest that BHs originally prepared for definitive *post-mortem* diagnosis and frozen for several years are generally suitable for RT-QuIC retrospective studies; however, the presence of one negative sample confirmed that long-term storage of BH, in the form of detergent lysate, may occasionally lead to a loss of RT-QuIC seeding activity.

We observed higher variability, lower max ThT fluorescence, and longer time to threshold in the type-2 sCJD compared to type-1 sCJD BH samples, suggesting that the PrP^Sc^ glycotype may affect the seeding potential. However, no effect of the sCJD glycotype alone on the seeding potential of CSF has been previously reported [13]. On the other hand, the higher seeding ability of the gCJD E200K samples, in comparison to sCJD BH samples, was in agreement with published CSF results [25]. Interestingly, the difference between the gCJD E200K and sCJD groups was not evident at the initial 5 × 10^−6^ dilution and manifested at higher BHs dilutions, especially at a dilution of 5 × 10^−8^.

A comparison of the time to threshold parameter demonstrated the difference between the gCJD E200K and sCJD groups at all dilutions. The positivity manifested much faster in the gCJD E200K group and the variability was very low compared to the sCJD group. Our data suggest that the seeding activity of gCJD E200K present in archived *post-mortem* BHs was higher than in sCJD samples; however, the cause of this difference has to be elucidated. The greatest variability of results was observed in the GSS group. This phenomenon has been described in the literature and was also evident in our archival samples [14,26]. On the positive side, all archived GSS BHs tested positive, even after long-term storage. All tested TSE samples with a positive result had maximal ThT fluorescence values safely above the threshold, except for the case of VPSPr; however, even this sample was clearly above the threshold [25].

In most of the cases included in our BH study, we also had archival *post-mortem* CSF samples. The main problem with the analysis of *post-mortem* CSF samples was the excessive protein concentration connected with inhibition of the RT-QuIC assay and false negative results. The high concentration of proteins present in *post-mortem* CSF has already been reported [27]. Simple dilution of the CSF samples allowed for detection of their seeding activity in almost all TSE samples, with 95.8% sensitivity and 100% specificity. A possible inhibitory effect has been described in a CSF sample with a total protein concentration higher than 0.45 g/L [28]. Tze How Mok et al. showed, using *post-mortem* CSF samples, that only 70 of 79 sCJD samples and 9 of 20 various inherited prion diseases were positive, using undiluted *post-mortem* CSF in RT-QuIC with bank vole prion protein as substrate [29]. The lower sensitivity could be due to the high protein concentration in the *post-mortem* samples. We did not observe inhibition of seeding up to the concentration of 2.5 g/L of total protein. Above this value, there was a significant reduction in the sensitivity of the assay. In our assay, the threshold determined from the diluted CSF negative controls was twice as high as the threshold from the undiluted controls. This phenomenon is important to consider when testing CSF samples, where excessive amounts of protein can lead to a false negative result. Furthermore, when the threshold for diluted samples is calculated using undiluted high protein negative controls, false positive results can occur. It must be noted that, despite great care and proper technique, the *post-mortem* sampling of ventricular CSF is inherently associated with a risk of CSF contamination by brain tissue. Although the level of contamination is likely to be negligible, its effect on RT-QuIC results cannot be ruled out, and the 100% sensitivity reached in our *post mortem* CSF study should be interpreted with caution.

To sum up the most important result of our validation study, we proved that archival *post-mortem* BHs or CSF samples can be easily analyzed by RT-QuIC, regardless of the storage duration and the detergents present in the storage buffer, without interfering with the assay. The second-generation RT-QuIC assay with shortened rSHa PrP(90–231) substrate utilized in our study was able to detect seeding activity in all types of human TSEs tested, including rare gCJD (D178N, R208H), GSS P102L, and VPSPr cases. Even though the number of samples in the experimental groups was relatively low, the observed differences in the max ThT fluorescence and time to threshold among the diverse TSEs suggest that further refinement of the RT-QuIC assay may lead to the development of diagnostic laboratory tools capable of distinguishing specific types of human prion diseases.

## 4. Materials and Methods

### 4.1. Ethics Statement

The study was reviewed and approved by the Ethics Committee of the Institute of Clinical and Experimental Medicine and Thomayer Hospital in Prague, Czech Republic (approval no. G-17-06-28).

### 4.2. Prion Disease Samples

All samples were provided by the National Reference Laboratory for Diagnostics of Human TSE/CJD, Thomayer University Hospital, Prague, Czech Republic. From sporadic patients, we selected three different cases from each sCJD type, according to the codon 129 polymorphism and glycotype (MM1, MV1, VV1, MM2, MV2, VV2).

In addition to this group, we included variably protease-sensitive prionopathy (VPSPr) as an unusual case of sporadic prionopathy (n = 1). From the group of genetic prion diseases, we included gCJD E200K (n = 15), Gerstmann–Sträussler–Scheinker syndrome with pathogenic mutation P102L in *PRNP* gene (GSS, n = 3), and gCJD D178N (n = 1) and gCJD R208H (n = 1) mutations. All BH samples were originally prepared for SDS-PAGE and western blot confirmation of TSE diagnosis. The frontal cortex tissue was homogenized in 10 volumes of lysis buffer (25 mM Tris HCl, pH 7.6, 0.5% TERGITOL NP405, 12 mM sodium deoxycholate) aliquoted, and the 10% BHs were stored in a deep freezer (−80 °C) until use in second-generation RT-QuIC.

The sample storage span was from 2 to 15 years. CSF samples were collected during the autopsy and stored in a deep freezer (−80 °C) for 2 to 15 years. As negative controls, we used BHs from corneal donors (n = 25) and from patients with other confirmed neurological disorders (OND, n = 28). OND samples (BH and CSF samples) were collected from patients with frontotemporal lobar degeneration with τ pathology (FTLD-tau, n = 5), frontotemporal lobar degeneration with TDP-43 inclusions (FTLD-TDP, n = 5), Alzheimer’s disease (AD, n = 5), Parkinson’s disease (PD, n = 5), Alzheimer’s disease with additional pathological signs (AD + others, n = 4), and Huntington disease (HD, n = 4) (Table 1).

### 4.3. Production of Recombinant Prion Protein for the RT-QuIC Assay

As a substrate in RT-QuIC reaction, we used truncated recombinant Syrian hamster PrP of residues 90–231 [rSHa PrP (90–231)]. Expression plasmid (pET41) was kindly provided by Professor Jiri Safar (Departments of Pathology and Neurology at Case Western Reserve University, Cleveland, Ohio, USA). We prepared rSHa PrP (90–231) as previously described [10]. Briefly, *E. coli* (Rosetta™ (DE3), Novagen, Merck KGaA, Darmstadt, Germany) bacteria harboring plasmid encoding rSHa PrP (90–231) under an inducible promotor were cultivated in LB media (Chloramphenicol/Kanamycin) overnight at 37 °C, 300 rpm. Protein expression was induced by Overnight Express™ Autoinduction System 1 (Novagen, Merck KGaA, Darmstadt, Germany). Bacteria were harvested and inclusion bodies were purified by BugBuster Master Mix (Novagen, Merck KGaA, Darmstadt, Germany). Inclusion bodies were dissolved in 8 M guanidine hydrochloride, and the protein was purified using the BioLogic LP Low-Pressure Liquid Chromatography System (Bio-Rad, Prague, Czech Republic) and a 100 mL column (GE Healthcare XK Chromatography Columns, Merck KGaA, Darmstadt, Germany) containing 54 g of Ni Sepharose^®^ 6 Fast Flow (Cytiva, Merck KGaA, Darmstadt, Germany). Binding of denatured PrP was carried out in denaturing conditions (100 mM Sodium Phosphate, pH 8.0; 10 mM TRIS; 6 M Guanidine HCl). The protein refolding step was carried out on a column by exchange of denaturing conditions (100 mM Sodium Phosphate, pH 8.0; 10 mM TRIS; 6 M Guanidine HCl) with physiological conditions (100 mM Sodium Phosphate, pH 8.0; 10 mM TRIS) by linear gradient (flow rate 2.3 mL/min, 280 min). Refolded rSHa PrP (90–231) was eluted using a linear gradient with imidazole (100 mM Sodium Phosphate, pH 5.8; 10 mM TRIS; 500 mM Imidazole) and dialyzed against 10 mM Sodium Phosphate (pH 5.8). The final concentration was determined by a spectrophotometer (Eppendorf, Prague, Czech Republic). The purity of the prepared recombinant protein was confirmed by SDS-PAGE and western blot (3F4 and 6D11 antibody) [30] and Coomassie staining. The quality and tendency of spontaneous aggregation for each preparation was tested by the second-generation RT-QuIC assay.

In order to decrease the costs of the rSHa PrP (90–231) preparation, we recycled Ni Sepharose. After each purification, the Ni Sepharose was stripped by EDTA (20 mM sodium phosphate, 0.5 M NaCl, 50 mM EDTA, pH 7.4), to remove all Ni^2+^, and recharged again by 0.5 column volume of 0.1 M NiSO_4_, according to the manufacturer’s instructions (Cytiva, Merck KGaA, Darmstadt, Germany). We used the Ni Sepharose up to three times, with no detectable effect on the quality of recombinant prion protein in the second-generation RT-QuIC assay.

### 4.4. Second-Generation RT-QuIC

The detection of prion seeding activity in samples was accomplished using the second-generation RT-QuIC assay [9,10]. All samples were run in quadruplicate. The assay was performed in Nunc™ MicroWell™ 96-Well Optical-Bottom Plates (Thermo Fisher, Prague, Czech Republic) in 100 µL reaction mix for each well (10 mM phosphate buffer, pH 7.4; 300 mM NaCl; 10 µM thioflavin T (ThT); 1 mM EDTA; 0.002% SDS; and 0.1 mg/mL of rSHa PrP (90–231)). For the testing of brain homogenates (BHs), 2 µL of BH were added to 98 µL of reaction volume. BH samples were diluted 5 × 10^−6^ up to 5 × 10^−9^ in PBS buffer containing 1× N-2 supplement (Gibco, Thermo Fisher, Prague, Czech Republic N-2 Supplement, 100×) and 0.1% SDS. CSF samples were tested undiluted or 10 times diluted in PBS (pH 7.4) and 15 µL of CSF sample were added into 85 µL reaction buffer. The reaction was carried out by FLUOstar Omega plate reader (BMG LABTECH GmbH, Ortenberg, Germany), undergoing repeating shaking cycles of 60 s (700 rpm, double orbital) and 60 s rest for 60 h at 55 °C. The fluorescence was measured every 15 min. Each test was analyzed by Mars software (BMG LABTECH GmbH, Ortenberg, Germany).

### 4.5. CSF Protein Concentration Measurement

Protein concentrations of CSF samples were measured by the Pierce BCA Protein Assay Kit (Thermo Fisher Scientific, Prague, Czech Republic) and Victor 3 multilabel plate reader (Perkin Elmer, PE systems, Prague, Czech Republic). To avoid exceeding the detection limit of the assay, the CSF samples were measured undiluted and 10 times diluted.

### 4.6. Data Analysis

To discriminate positive and negative samples, the threshold value (of fluorescence units, in figures expressed as arbitrary units; AU) was determined using negative controls. Quadruples of 28 OND control samples were analyzed by RT-QuIC and the average maximum fluorescence (S max) was calculated for each sample. The threshold was set using the average of S max values of all negative samples plus five times the standard deviation. The thresholds were determined separately for BH and CSF samples. Samples with average S max above the threshold were concluded as positive when at least two wells out of four were positive. In case of just one positive well, a second round of the test was performed. Samples were assigned a positive result when at least two out of eight wells were positive (counting the results of the first and second rounds of the test together) [10].

## Figures and Tables

**Table 1:** Demographic data and diagnostic investigation results in tested group of prion diseases cases and control groups.

Group	Type	n (BH)/(CSF)	AST (Years ± sd)	Mean Age (Years)	t-tau (Pos./Tested)	14-3-3 (Pos./Tested)
**Sporadic TSE**	MM1	3/3	2 ± 0	71	3/3 (100%)	2/3 (66.7%)
	MM2	3/3	6.7 ± 0.6	65	1/1 (100%)	2/3 (66.7%)
	MV1	3/3	3.3 ± 0.6	63	2/2 (100%)	1/2 (50%)
	MV2	3/3	4.3 ± 2.5	70	3/3 (100%)	2/3 (66.7%)
	VV1	3/3	2 ± 0.8	64	3/3 (100%)	2/3 (66.7%)
	VV2	3/3	6.3 ± 3.6	64	2/2 (100%)	2/2 (100%)
	VPSPr (VV)	1/0	2 ± 0	73	NT	NT
**Genetic TSE**	E200K	15/3	9 ± 3.5	61	7/8 (87.5%)	6/12 (50%)
	GSS	3/3	8 ± 3	48	2/2 (100%)	0/2 (0%)
	D178N (MV)	1/0	13 ± 0	59	NT	1/1 (100%)
	R208H (VV)	1/0	10 ± 0	62	1/1 (100%)	1/1 (100%)
**OND**	FTLD-tau	5/5	3.2 ± 2.2	71	0/3 (0%)	0/3 (0%)
	FTLD-TDP	5/5	3.6 ± 1.7	64	0/2 (0%)	0/3 (0%)
	AD	5/5	4.6 ± 2.3	70	NT	1/2 (50%)
	AD + others	4/4	2 ± 0	79	1/1 (100%)	NT
	Huntington dis.	4/5	5.8 ± 2.2	72	0/1 (0%)	NT
	PD	5/5	6 ± 2.9	70	1/1 (100%)	NT
**Healthy corneal donors**	-	25/0	-	-	NT	NT

AST, average storage time (homogenate with detergents in deep freeze −80 °C); FTLD-tau, frontotemporal lobar degeneration with τ pathology; FTLD-TDP, frontotemporal lobar degeneration with TDP-43 inclusions; AD, Alzheimer’s disease; AD + others, Alzheimer’s disease with additional pathological signs; PD, Parkinson’s disease; NT, not tested.

## Data Availability

Data are provided within the article.
